# Does trauma team activation associate with the time to CT scan for those suspected of serious head injuries?

**DOI:** 10.1186/1749-7922-8-48

**Published:** 2013-11-18

**Authors:** Alma Rados, Corina Tiruta, Zhengwen Xiao, John B Kortbeek, Paul Tourigny, Chad G Ball, Andrew W Kirkpatrick

**Affiliations:** 1 Regional Trauma Services, Foothills Medical Centre, University of Calgary, 29 Street, Calgary, NW 1403, Alberta; 2 Departments of Surgery, Foothills Medical Centre, University of Calgary, Calgary, Alberta; 3 Critical Care Medicine, Foothills Medical Centre, University of Calgary, Calgary, Alberta; 4 Radiology, Foothills Medical Centre, University of Calgary, Calgary, Alberta; 5 Emergency Medicine, Foothills Medical Centre, University of Calgary, Calgary, Alberta

**Keywords:** Trauma triage, Trauma activation, Trauma human factors, Traumatic brain injury, Diagnostic imaging

## Abstract

**Background:**

Traumatic brain injury (TBI) constitutes the leading cause of posttraumatic mortality. Practically, the major interventions required to treat TBI predicate expedited transfer to CT after excluding other immediately life-threatening conditions. At our center, trauma responses variably consist of either full trauma activation (FTA) including an attending trauma surgeon or a non-trauma team response (NTTR). We sought to explore whether FTAs expedited the time to CT head (TTCTH).

**Methods:**

Retrospective review of augmented demographics of 88 serious head injuries identified from a Regional Trauma Registry within one year at a level I trauma center. The inclusion criteria consisted of a diagnosis of head injury recorded as intubated or GCS < 13; and CT-head scanning after arriving the emergency department. Data was analyzed using STATA.

**Results:**

There were 58 FTAs and 30 NTTRs; 86% of FTAs and 17% of NTTRs were intubated prehospital out of 101 charts reviewed in detail; 13 were excluded due to missing data. Although FTAs were more seriously injured (median ISS 29, MAIS head 19, GCS score at scene 6.0), NTTRs were also severely injured (median ISS 25, MAIS head 21, GCS at scene 10) and older (median 54 vs. 26 years). Median TTCTH was double without dedicated FTA (median 50 vs. 26 minutes, p < 0.001), despite similar justifiable delays (53% NTTR, 52% FTA). Without FTA, most delays (69%) were for emergency intubation. TTCTH after securing the airway was longer for NTTR group (median 38 vs. 26 minutes, p =0.0013). Even with no requirements for ED interventions, TTCTH for FTA was less than half versus NTTR (25 vs. 61 minutes, p =0.0013). Multivariate regression analysis indicated age and FTA with an attending surgeon as significant predictors of TTCTH, although the majority of variability in TTCTH was not explained by these two variables (R² = 0.33).

**Conclusion:**

Full trauma activations involving attending trauma surgeons were quicker at transferring serious head injury patients to CT. Patients with FTA were younger and more seriously injured. Discerning the reasons for delays to CT should be used to refine protocols aimed at minimizing unnecessary delays and enhancing workforce efficiency and clinical outcome.

## Background

All trauma systems need to define the optimal criteria with which to activate full trauma responses in order to respond to the immediate clinical needs of the critically injured. Thus, the American College of Surgeons Committee on Trauma (ACS COT) has defined guidelines to guide prehospital triage to trauma centers [1]. Building on these guidelines, many centers recognize the need for two or three tiered activation criteria to more efficiently manage hospital and human resources [2-8]. Many systems including our own, require the immediate or urgent presence of attending trauma surgeons as their “highest level” response. Of the various criteria used to initiate full trauma activations, severe head injuries denoted by a depressed Glasgow Coma Scale (GCS) have long been the most controversial at our institution and the most problematic in terms of adherence to protocols and standards. Routine trauma quality assurance (QA) activities in our center note that this criterion represents the majority of failures to activate the trauma team [9]. While trauma surgeons from a general surgery specialty practically do not operate on severe head injuries it is perceived that they both contribute to resuscitative care and expedite the work-up. However, there is limited information regarding the time factors and efficiency of different trauma systems in triaging and optimizing the prompt attainment of CT imaging in the critically injured [10]. This prompted us to review the association between the type of trauma response and the efficiency of obtaining a CT scan in seriously head injured patients.

## Methods

The Alberta Health Services Calgary Region (AHSCR) is a fully integrated, publicly funded health system that provides virtually all medical and surgical care to the residents of the city of Calgary and a large surrounding area including smaller towns and communities (population ~ 1.2 million). In the AHSCR, adult trauma services are regionalized to the Foothills Medical Centre (FMC), and pediatric trauma services (age mandate ≤14 years) to the Alberta Children’s Hospital. These are the only accredited tertiary trauma care centers providing trauma services for Southern Alberta, Canada (~35% of the population of the Province of Alberta). Patients may also be transported to Calgary from trauma care services in neighboring provinces.

At FMC, full trauma activations (FTAs) involve an expedited response by an attending trauma surgeon and trauma team (TT), residents from critical care medicine, respiratory therapists, and other dedicated trauma resources including anesthesia and the operating room, in addition to emergency physicians and nurses who are the typical responders to initial non-trauma team responses (NTTR) (Table 1). Patients with an initial NTTR are often seen after the initial assessment by the emergency medicine team in the format of a trauma consult by the TT if admission or ongoing care is required. A FTA may be initiated by the emergency physician based on changing patient status, updated prehospital information, or clinical judgment. The response performance of trauma personnel is a trauma quality assurance audit filter and is assessed and reported annually in the Trauma Services Annual Report noting that recent audit revealed the attending trauma surgeons are typically always present within 20 minutes at a FTA [9].

**Table 1:** Alberta health services - Calgary Region trauma activation criteria

1.	Shock defined by BP systolic < 90 mmHg or Temperature ≤ 30°C
2.	Patient intubated for respiratory compromise/airway obstruction
3.	Patient with GCS ≤ 8
4.	Gunshot wound to the head, neck, or torso
5.	Need for blood transfusion *en route* to hospital or in the ED

In order to assess the efficiencies and human resource implications of trauma activations not focusing on traditional thoracoabdominal injuries, a retrospective review of trauma patient resuscitations with head injuries requiring intubation or with a GCS < 13 in whom a CT scan was obtained. Patients were identified from the FMC Trauma Registry as having been admitted between April 01 2008 and March 31, 2009. To qualify for the trauma registry a patient must have an Injury Severity Score (ISS) > 12 and be admitted to the trauma centre or die in the emergency department of the trauma centre.

From the eligible cohort (186 TBI patients who met the inclusion criteria), a convenience sample of 101 charts was selected by medical records for review. Demographic data reviewed included age, gender, emergency department (ED) admission date, ED admission time, injury description, Maximum Abbreviated Injury Scale (MAIS) Head, Injury Severity Score (ISS), scene GCS, trauma centre GCS, patient intubation status at the time of the GCS was calculated, whether FTA was activated, time of trauma team activation, trauma surgeon, intensive care unit (ICU) admission, ICU length of stay (LOS), and discharge status. The following data was collected directly from the charts: whether patient had a CT done at previous hospital, arrival time of trauma surgeon at FTA, CT head date and time, picture archiving and communication (PACS) time of CT head, electronic medical record time of CT Head, whether there was a reason for CT delay, and if there was a reason for delay then which interventions were done, interventions date, interventions time, and any comments about the patient. We initially sought to study the times until completion of the CT head. However review of the time imprints embedded with the CT images in PACS was found to be non-sensical clinically, and a subsequent review of the electronic clocks in the CT scanners found them to be significantly inaccurate. Thus, the charted time the patient left the trauma bay for the CT scanner was used instead. The “Time from ED admission to CT head (TTCTH-unqualified)” was defined as the unqualified number of minutes from ED admission until the patient left for the CT scan. The “Time in ED after airways were secure (TTCT-after airways secure)” was defined as either the time in the ED until leaving for CT head if intubated pre-hospital or never intubated, or as the time in the ED after ED intubation until leaving for CT head. For those re-intubated in ED, the time from re-intubation until leaving for CT was used for this designation. The “Time in the ED after intubation or procedure (TTCTH-after any procedure)” was defined as the time in the ED after any required procedure was performed including any of ED intubation, chest tube insertion, or arterial or central venous line insertion, and Focused Assessment with Sonography (FAST). If the time of the procedure was unavailable, or if no procedure was required, this time was measured from arriving in the ED until leaving for CT head. We also separately examined the TTCTH in patients who had no interventions of any type in the ED (TTCTH-no interventions), the TTCTH excluding patients who required intubation or re-intubation for misplaced endotracheal tubes in the ED (TTCTH-exclude intubation), and the TTCTH including only patients intubated (pre-hospital or in the ED) (TTCTH-intubation only).

The data were analyzed using STATA (version 9.2, College Station, Texas) and presented as medians with interquartile ranges (IQR) for non-normally distributed variables. Medians were compared using the Mann-Whitney U test, categorical data were analyzed by Fisher’s exact test. To identify independent factors associated with the time to CT Head a multiple linear regression model was developed, using backward stepwise variable elimination. Statistically significant differences were defined as a *p* value < 0.05.

## Results

One hundred and one (101) eligible patients’ charts were reviewed. Thirteen (13) patients were excluded from the final analysis as seven patients had CT head done at a referring hospital, four had missing times to CT, one was not trauma patient and one did not have a TBI leaving 88 records for analysis. Fifty-eight (58) patients had a FTA, and 30 had a NTTR. Patients in the FTA group were younger (median age 26 vs 54 years), higher median ISS (29 vs 25, p = 0.007), and lower scene GCS score (6 vs 10, p = 0.08) than the NTTR patients, with the majority being intubated prehospital. Table 2 shows the characteristics of the two groups. The actual time of the trauma team activation was recorded in only 21 (36%) of activations, but all had ER admission time recorded. In 11 cases the FTA was prior to emergency department (ED) admission, in 8 it was coincident with ED admission, and in 2 after admission. Thus the median time to FTA was 1 minute before ED admission with an average time of 5.5 minutes noting one outlying activation 164 minutes after ED admission.

**Table 2:** Patient characteristics in resuscitative groups (FTA and NTTR)

**No. of patients**		**FTA**	**NTTR**	**p value**
**N = 88**		**(n = 58)**	**(n = 30)**	
Age (y)	median (IQR)	26 (21–46.5)	54 (25.5-76.5)	0.0017
	mean ± SD	35 ± 18	51 ± 24	
Male gender		46 (79%)	22 (73%)	0.6
ISS	median (IQR)	29 (23.5-41.5)	25 (17–29)	0.0071
	mean ± SD	32 ± 11	25 ± 7.5	
MAIS Head,	median (IQR)	16 (16-25)	20.5 (16-25)	0.5
	mean ± SD	19 ± 6	20 ± 6	
GCS at scence,	median (IQR)	6.0 (3.0-12.0)	10.0 (5.75-13)	0.08
Intubated prehospital		50 (86%)	5 (17%)	<0.0001
Intubated in ED^1^		5 (8.6%)	11 (37%)	0.0026
No. pts with reason for delay to CT^2^		30 (52%)	16 (53%)	1
No. pts with ED Interventions^3^		27 (47%)	14 (47%)	0.9
TTCTH-unqualified				
Time from ED adm to CT (min), median (IQR)		26 (19.5-36.5)	49.5 (32–80.5)	<0.001
TTCTH-after airways secure (min)^4^		25.5 (17.5-35)	38 (27.5-78)	0.0013
TTCTH-after any procedure (min)^5^		22.5 (16–32)	34.5 (24–78)	0.0007
ICU Admissions		43 (74%)	13 (43%)	0.006
ICU LOS^6^, median (IQR)		3 (1–10.5)	3 (1-9)	0.7
In-hospital death, n (%)		16 (27.5)	12 (40)	0.334

^1^ one FTA pt and 2 NTTR pts were reintubated in ED.

^2^ delay to CT could be caused by an intervention in ED or by non-procedure factors.

^3^ interventions in ED include: intubation,chest tube,FAST, arterial line,resuscitation,etc.

^4^ Time in the ED after intubation until CT or from ED admission until CT if intubated prehospital or never intubated (includes prehospital intubated, intubated in ED, never intubated).

^5^ Time of intervention done in ED was not found in all cases, thus time from ED admission to CT was used.

^6^ LOS, length of stay in days.

Patients who presented during FTA (n = 58) had a significant shorter time to CT head compared with patients evaluated with a NTTR (n = 30) (TTCTH-unqualified 26 min [IQR = 19.5-36.5] vs 49.5 min [IQR = 32-80.5]; p <0.0001) (Table 2). As expected, there was an association between trauma team activation and pre-hospital intubation, with a coefficient of correlation r =0.6. Using CT head as the dependant variable, a multiple linear regression analysis with age, ISS, MAIS head, ED intubation, trauma team activation designation, pre-hospital intubation, and requirement for any ED intervention as predictors was performed (Table 3). Backward stepwise variable elimination identified age and trauma team activation as significant predictive factors influencing reduced time to CT head. Time to CT Head was predicted to be 1.8 minutes lower per one unit increase in FTA; however, this group of variables does not fully explain the variability in time to CT Head (R² = 0.33).

**Table 3:** Multiple linear regression: predictors of time to CT Head

**Initial independent Variables**	**Coefficients**	**Std. Err**	**t**	**p > |t|**	**[95% Conf. interval]**	
Age	0.0070221	0.0028789	2.44	0.017	0.0012917	0.0127525
MAIS Head	-0.0156356	0.0100677	-1.55	0.124	-0.0356748	0.0044067
ISS	-0.0000174	0.0066377	-0.00	0.998	-0.0132293	0.0131945
Pre-hospital intubation	-0.2816034	0.1642582	-1.71	0.090	-0.6085512	0.0453443
Trauma team activation	-0.4942918	0.1754433	-2.82	0.006	-0.8435029	-0.1450807
ED intubation	-0.2740521	0.1862904	-1.47	0.145	-0.644854	0.0967497
ED intervention	0.1633863	0.1372994	1.19	0.238	-0.1099013	0.4366739
Predictor Variables of time to CT Head	Coefficients	Std. Err	t	p > |t|	[95% Conf. interval]	
Age	0.00617341	0.0028299	2.18	0.032	0.0005458	0.0118009
Trauma team activation	-0.6133904	0.1255942	-4.88	0.000	-0.8631482	-0.3636326

Although the majority of cases were intubated prehospital, 11 (37%) of the NTTR pts vs. 5 (9%) FTA pts were intubated after arriving in ED. The TTCTH was shorter for FTA (median 25 vs. 45 minutes for NTTR) but limited by the few patients intubated in ED. With intubation after arriving in ED being the top cause of delays to CT for NTTRs, we examined only those patients without any need for ED intubation to ensure more similarity between the two groups. The TTCTH-exclude intubation was 27 versus 55 minutes (p =0.0015) favoring FTA (Table 4). For the whole group of patients (intubated pre-hospital, intubated in ED, or never intubated) the TTCTH-after airways secure was 26 minutes versus 38 minutes (p =0.0013) in favor of FTA (Table 2). Just over half of each group had documented resuscitative procedures before being taken to CT (FTA = 47%, NTTR = 47%). For all patients, the TTCTH-after any procedures was 23 versus 35 minutes (p =0.0007) favoring FTA (Table 2), and the TTCTH-no interventions was 25 versus 61 minutes (p =0.0013) favoring FTA as well (Table 5). For patients intubated pre-hospital or in ED the time from arriving in the ED until CT was also shorter for FTA group (median 26 versus 45 minutes, p =0.002). Although a specific review of TTCTH-unqualified for all patients with pre-hospital intubation was limited by the few patients in NTTR (n = 5), this group took 33 minutes compared to 26 minutes in FTA (n = 50). All comparison of times is summarized in Table 6.

**Table 4:** Times to CT head excluding patients with any need for emergency department intubation (or re-intubation)

	**FTA**	**NTTR**	**p value**
**No.of pts (72)**	**(n = 53)**	**(n = 19)**	
Age, median (IQR)	26 (21–46.5)	65 (43–77)	<0.0001
Gender, male	42 (79%)	12 (63%)	0.2
ISS, median (IQR)	29 (23.5-41.5)	25 (16–29)	0.0032
MAIS Head, median (IQR)	16 (16-25)	16 (16-25)	0.7
No.pts preintubated	49 (92%)	3 (16%)	<0.0001
No.pts who underwent any type of procedure in ED	22 (42%)	3 (16%)	0.0526
TTCTH-exclude intubation			
Time from ED adm to CT, median (IQR)	27 (19–36.5)	55 (30–107)	0.0015

**Table 5:** Times to CT head for patients with no emergency department interventions

	**FTA**	**NTTR**	**p valve**
**No. of pts (47)**	**(n = 31)**	**(n = 16)**	
Age, median (IQR)	26 (20–48)	67 (45.5-77)	0.0005
Gender, male	22 (71%)	11 (69%)	1
ISS, median (IQR)	29 (20–41)	25 (16–25.5)	0.02
MAIS Head, median (IQR)	16 (16-25)	20.5 (16–25)	0.7
No.pts preintubated	30 (97%)	3 (19%)	<0.0001
TTCTH-no interventions			
Time from ED adm to CT, median (IQR)	25 (17–32)	60.5 (30–123.5)	0.0013

**Table 6:** A summary of the times from arriving in the ED until CT head for different subgroups of patients

	**FTA**	**NTTR**	**p value**
**No. of Pts**	**n = 58**	**n = 30**	
Median min. (IQR)	26 (19.5-36.5)	49.5 (32–80.5)	<0.001
Intubated	n = 50	n = 5	sample too small
Pre-hospital			
Median min (IQR)	26 (18.5-36.5)	33 (25–74.5)	
Intubated or	n = 5	n = 11	sample too small
Re-intubated in ED	*1 pt reintubated	*2 pts reintubated	
Median min (IQR)	25 (20.5-32)	45 (42–62)	
Pts w/o ED	n = 53	n = 19	0.0015
Intubation			
Median min (IQR)	27 (19–36.5)	55 (30–107)	
Pts w/o ED	n = 31	n = 16	0.0013
Intervention			
Median min (IQR)	25 (17–32)	60.5 (30–123.5	
Intubated	n = 54	n = 14	0.0002
Pre-hospital or in ED			
Median min (IQR)	26 (19–36.5)	45 (36–67.5)	

## Discussion

Many combinations of mechanistic, anatomic, physiologic, and demographic criteria, generally adapted from the Field Triage Decision scheme of the ACS COT [1], have been adopted by numerous investigators and organizations to guide the field triage of the trauma patient [1,4-7]. The ideal triage system to manage competing clinical needs with practical resource management remains elusive. Such an ideal system would equally match the severity of injury and resources required for optimal care with the optimal facilities, personnel, and response criteria [1.5]. One of the most limited resources is that of the responding trauma surgeons themselves. In systems that require the immediate or urgent presence of attending trauma surgeons this “non-surgical” task may exacerbate what has been perceived to be a crisis in trauma surgery human resources [4,11-14].

Contemporary initiatives have focused on identifying patients requiring specific emergency department procedures or operative interventions to define which of the many potential triage criteria are valuable or not [5]. In addition to identifying the need for a procedure, we suggest that significantly decreasing the delay until a critically injured patient with a potentially treatable space-occupying lesion detected on CT scanning is another critical aspect of full trauma activation. This needs to be evaluated as a process outcome. Simply put, time is brain. The duration of brain herniation before surgical decompression influences outcomes for acute epidural hematomas [15,16], and as such, obtaining urgent CT scans is typically a requisite part of brain injury preoperative resuscitation. As we believe that expediting the resuscitative and diagnostic workup of the critically injured is important to their outcome, we have included intubated head injuries as an activation criterion for full trauma activation.

CT scanning is considered the reference standard for diagnosing most traumatic injuries in the acutely injured patient [17-23] and specifically for detecting post-traumatic intra-cranial lesions [24,25]. Despite the primacy of CT scanning as the preferred definitive imaging modality however, there is limited information regarding the time factors and efficiency of different trauma systems in triaging and optimizing the prompt attainment of this imaging modality in the critically injured [10]. In one of the few reviews of CT efficiency, Fung Kon Jin and colleagues [10] found that the median start time in a high-volume “stream-lined” level-1 American trauma center for a severely injured cohort (median ISS 18) was 82 minutes, with the median time from arrival until completion of the diagnostic trauma evaluation being nearly 2 hours (114 minutes). The relevance of this time may be increased by noting that the mean time to CT head for non-traumatic neurological emergencies in a tertiary care academic institution that prioritized CT scanning for potential stroke over all other emergency department patients except trauma was either 99 or 101 minutes, depending on whether there were competing trauma activations [26].

In terms of patients with severe TBI, efforts to expedite diagnostic imaging in general include the introduction of CT scanning directly into the trauma room. Such a scanner in Amsterdam has reduced the time until completion of CT diagnostic imaging to 79 minutes in a cohort in whom the majority had an ISS < 16 [27]; to 23 minutes in a German CT equipped resuscitation room caring for a population with a mean ISS of 24 [28]; and to 12 minutes in an Austrian cohort (mean ISS = 27) in whom scanning was started immediately after admission. In the Austrian cohort a systolic BP > 70 mmHg was considered sufficient for CT scanning without cardiac arrest [25].

Based on our review however, we believe another strategy is to continue to retain the category of severe TBI as a criterion for full trauma team activation that is likely applicable to similar institutions. At least in our institution this associates with specifically decreased time to obtain head CT scans in those with severe head injuries, and mandates the presence of a surgeon to facilitate invasive interventions. Several groups have confirmed that a GCS < 8 was associated with high mortality [6,8], and such patients were 100 times more likely to die, 23 times more likely to require ICU, and 1.5 times more likely to need an operation among trauma patient admissions [6]. Although we cannot significantly prove in-hospital mortality, the designation of a trauma as requiring “activation” was associated with a 1.8 minute decrease per “unit” of activation in TTCTH statistically. We perceive this to be associated with the dedicated presence of the trauma surgeon as the team leader and to a general “entitlement” of the patient to all other human and technical resources available in our hospital resulting in markedly short durations to CT. Noting that a reported delay in NTTRs was “CT unavailable” reinforces this presumption. However, this study was not designed to compare the efficacy between a non-surgeon and a surgeon led trauma team activation.

There are limitations of this review that are both generic to retrospective reviews in general and specific to our data. Firstly, this non-randomized methodology can only note the association between FTAs at our institution and expedited transfers to CT scan and cannot delineate which specific factors or procedures were responsible. Further, we do not have exact data on the responding time for the trauma surgeons for all FTAs. There were further distinct differences between the two groups of patients with a greater need for definitive airway interventions in the non-FTA group. However, even after looking specifically at the TTCTH after secure airway control or after the performance of required resuscitative interventions it was still distinctly quicker in the FTA group. Finally we were surprised to realize that the time imprints embedded directly onto radiological images were inaccurate which has obvious implications for quality assurance and medico-legal review. We now regularly check for accuracy in this regard.

## Conclusions

Full trauma activations involving attending surgeons were quicker at transferring seriously head-injured patients to CT. Patients with FTA were younger, higher ISS, lower scene GCS, and more often intubated in the pre-hospital setting. Discerning the reasons for delays to CT should be used to refine protocols aimed at minimizing unnecessary delays and maximizing workforce efficiency.

## Abbreviations

ACS COT: American College of Surgeons Committee on Trauma; BP: Blood pressure; CT: Computed tomography; ED: Emergency department; FAST: Focused assessment with sonography; FMC: Foothills Medical Centre; FTA: Full trauma activation; GCS: Glasgow coma scale; ICU: Intensive care unit; ISS: Injury severity score; IQR: Interquartile ranges; LOS: Length of stay; MAIS: Maximum abbreviated injury scale; NTTR: Non-trauma team response; PACS: Picture archiving and communication system; TBI: Traumatic brain injury; TTCTH: Time to CT head.

## Competing interests

The authors declare that they have no competing interests.

## Authors’ contributions

Study concept and design: AK, AR; Acquisition of data: AR, CT, AK; analysis and interpretation of data: AR, CT, AK, ZX, CB, PT; drafting of the manuscript: AK; critical revision of the manuscript: AK, ZX, CB. All authors read and approved the final manuscript.
